# Systematic review of the effect of insertion torque on locking screw performance

**DOI:** 10.1530/EOR-2025-0134

**Published:** 2026-03-02

**Authors:** James W A Fletcher

**Affiliations:** ^1^ Department for Health, University of Bath, Bath, UK; ^2^ Department of Orthopedic Surgery and Traumatology, Groupement Hospitalier de L’Ouest Lémanique, Nyon Hospital, Nyon, Switzerland

**Keywords:** screw, locking, torque, fixation

## Abstract

**Purpose:**

- Locking plates are well established as a powerful plating concept, especially in low-density bone. Construct strength is dependent on the fixation between the screw head and the plate; however, the influence of variations in the torque used to engage the screw head into the plate hole is unclear. The aim of this study was to systematically review the effect of insertion torque on the performance of locking screws.

**Methods:**

- A systematic review was performed with electronic searches of four databases using free and MeSH search terms. Systematic evaluation and data extraction was performed up to 2 April 2025. The principal outcome was the difference in failure load for the locking plate construct following variation of the insertion torque.

**Results:**

- The initial search identified 894 potentially relevant studies, of which six were eligible for inclusion. All were *in vitro*, biomechanical studies. Both stainless steel and titanium plates were tested. The former material showed no consistent performance increase with increased insertion torque in either pushout or cantilever testing. Titanium plates showed an increased pushout force and cantilever strength with more torque for some, but not all designs. Once screw head engagement in the plate hole was achieved, further torque was not clearly beneficial.

**Conclusion:**

- Increased insertion torque, beyond that required to seat the screw head, did not consistently show performance benefits. Most studies were underpowered and may not be representative of clinical failures. Further work to define optimum torque ranges for locking plate systems is required.

## Introduction

Locking plates have greatly enhanced orthopaedic treatment options. Numerous advantages of locking plate designs over non-locking have been demonstrated, such as preservation of periosteal blood supply, uniform load distribution, reduced infection rate (1), reduced screw loosening and increased screw pull-out resistance, especially in low-density bone (2, 3, 4, 5). Locking designs rely on the interaction between the screw head and the plate, to provide stability that is not contingent on the compression of the plate on the bone (1). Thus, the success of a locking construct is dependent on the fixation between the screw head and the locking hole of the plate. In turn, this fixation is affected by the insertion torque used on the screw head, driving the head into the receiving hole. It is postulated that ‘lower’ torque values might lead to a weaker construct and screw head disengagement at lower forces, whilst ‘higher’ torque values could generate sufficient deformation to make subsequent removal harder (6, 7, 8, 9, 10), with or without any biomechanical benefit. However, it is unclear what torque values are ‘lower’ and ‘higher’. Some implant manufacturers recommend insertion torques for their screws (11, 12, 13, 14), whilst other manufacturers provide only qualitative advice on the insertion technique (15). Further to this, the impact from not achieving or conversely from exceeding the recommendations is not clear. Torque limiters are available to help achieve a target torque; however, they can undertighten compared to the desired torque, especially when used with a powered driver rather than manual (16), or can overshoot the target (17).

Identifying an optimum and minimum torque for locking screw insertion or the general effect the insertion torque has on screw performance would greatly benefit surgical practice and could influence further locking plate designs and insertion methods. Accordingly, this study aimed to examine how insertion torque affected the performance of locking screws by conducting a systematic review of the available literature.

## Methods

A systematic review was performed in line with the Preferred Reporting Items for Systematic Reviews and Meta-Analyses (PRISMA) guidance (18). The search strategy employed free and Medical Subject Headings (MeSH) search terms and a combination of keywords relating to insertion torque for locking screws (“locking screw” AND “insertion torque”; “locking” AND “insertion”; “locking” AND “torque”; “locking plates” AND “insertion torque”). There were no restrictions on publication dates. MEDLINE, EMBASE, Web of Science and the Cochrane Library electronic databases were searched up to 2 April 2025. Only articles in English were considered. Studies were included that involved any variation in the insertion torque and tested screw insertion. All bone models were included (human and animal (both *in vivo* and cadaveric), and artificial). Exclusion criteria were failure to provide results due to changes in insertion torque – insertion torque was not a variable in the experiments – and non-axial insertion; studies that involved non-axial or off-axis screw insertion were excluded to avoid introducing additional confounding factors, such as angular mismatch, variable thread engagement and changes in the contact interface geometry, all of which could obscure the isolated effect of insertion torque on screw–plate performance. Reference lists of the included manuscripts were manually scanned for any relevant additional studies. The principal outcome of interest was the difference in the failure load for the screw plate construct with different insertion torques.

## Results

**Figure 1:**
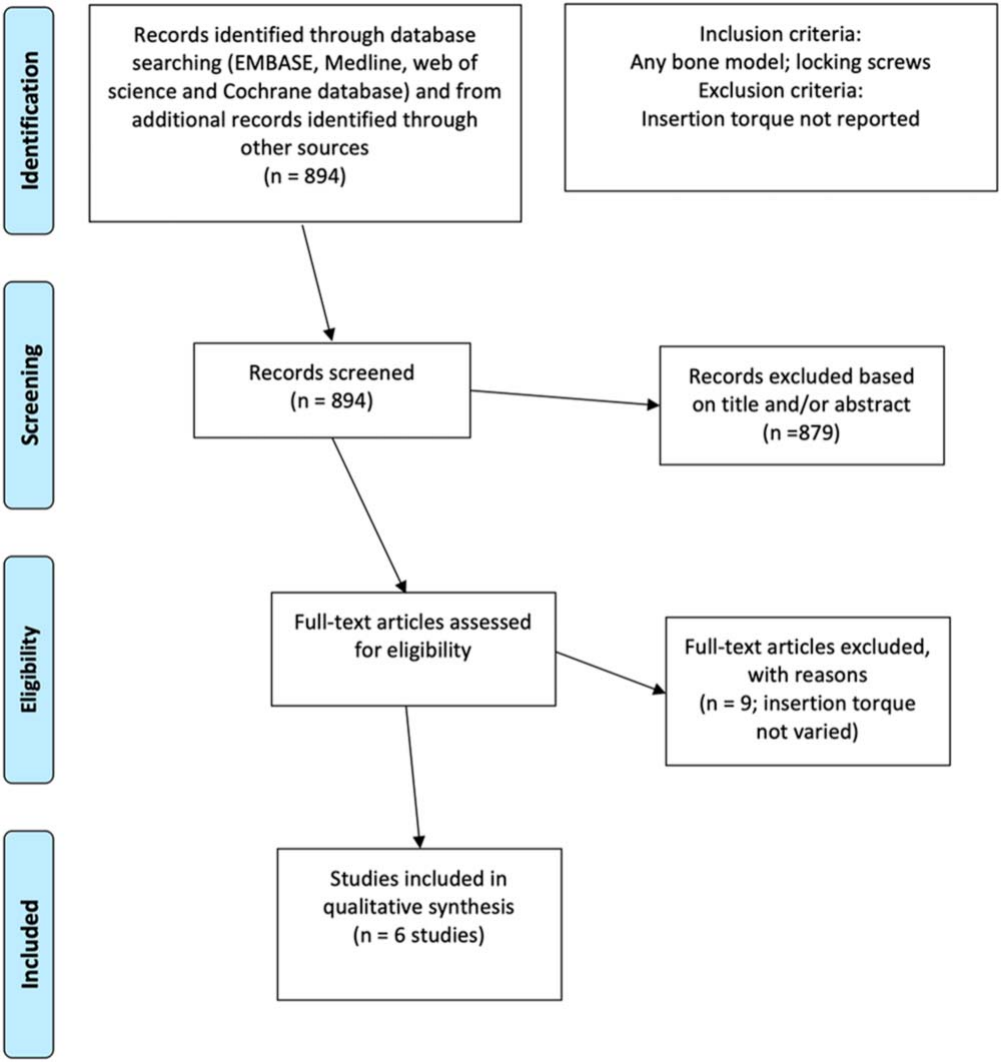
PRISMA flow chart.

**Figure 2:**
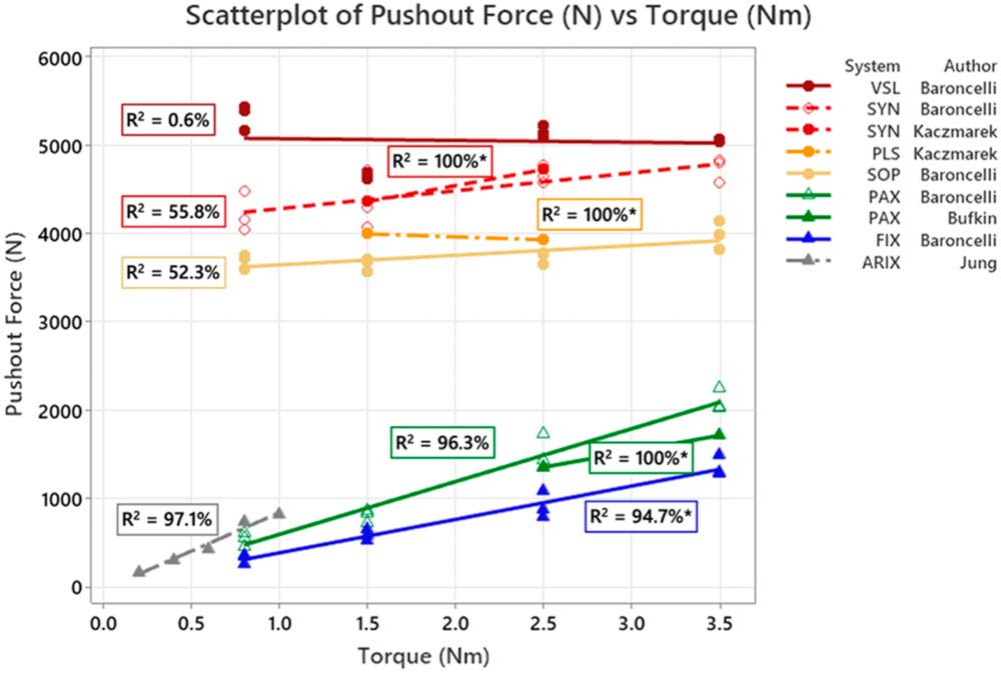
Pushout force compared to insertion torque for stainless steel (circles) and titanium (triangles) plates for ø3.5 and ø2.0 mm screws. The stainless steel plates have a thread-in-thread locking mechanism, whereas the titanium plates (with the exception of the ø2.0 mm ARIX screws) did not have a thread-in-thread mechanism. The gradient from red to yellow of the stainless steel plates indicates the number of threads from the most engagement (7 head threads and 3 hole threads of the VSL) to the least of 2 threads on the screw and 2 on the screw hole of the SOP system. The titanium system had threaded screw holes. The PAX system had a threaded head that cut a path, whereas the FIX system had neither a threaded screw head nor a screw hole relying on a bushing mechanism. The dashed trend lines indicate an interrupted thread, the equidistant dashes indicate a combi-hole (SYN), and the long and short dashes indicate interruptions for variable-angle locking (PLS and ARIX).

**Figure 3:**
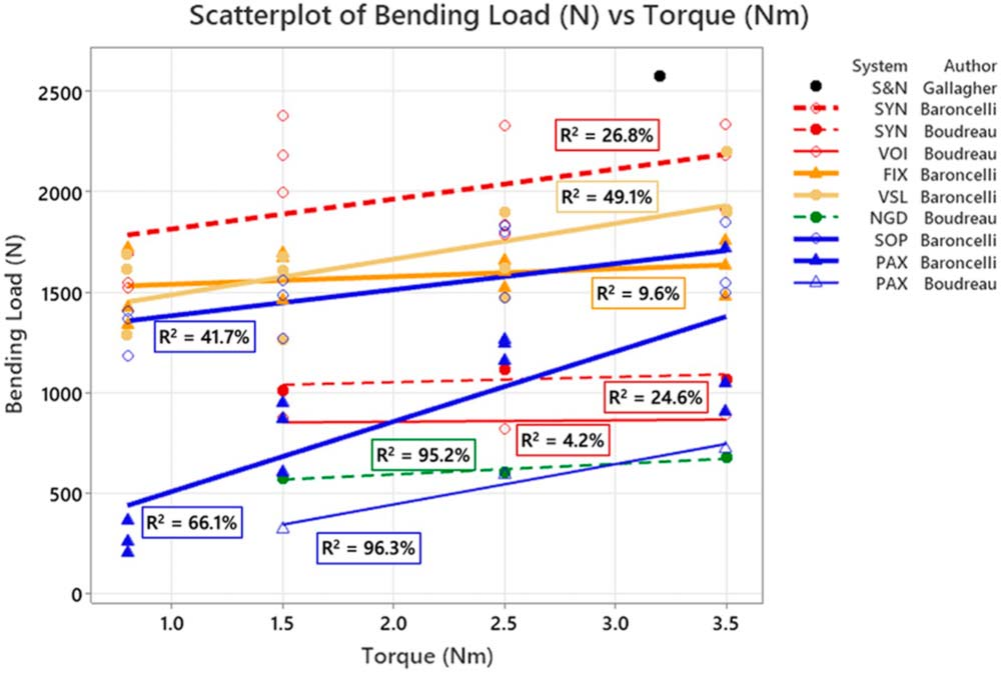
Cantilever bending failure force compared to insertion torque for stainless steel (circles) and titanium (triangles) plates. The gradient in colours follows the core diameter of the screws: red = ø2.9 mm, orange = ø2.8 mm, yellow = ø2.7 mm, green = ø2.6 mm and blue = ø2.4 mm. The trend line width indicates the difference between authors and test methods: Baroncelli = thicker trend lines; Boudreau = thinner trend lines, also evident in the lowest four values at 3.5 Nm. The dashed trend lines indicate an interrupted thread (SYN and NGD).

Our literature searches identified 894 potentially relevant studies (Fig. 1). A further review of the titles and abstracts reduced the potentially relevant studies to 15. On full reading, nine were excluded as insertion torque was not a variable and/or screw insertion was not tested. Six remaining articles were included in the review (7, 8, 10, 19, 20, 21). The findings of the six included studies are summarised in Table 1, with the available biomechanical values for pushout testing and cantilever testing shown in Figs 2 and 3, respectively.

**Table 1:** Effect of insertion torque on the performance of locking screw reported in the studies included in this review. Titanium plates are Ti-6Al-4V, and stainless steel plates are 316L. Statistical significance with value or non-significance (NS) is indicated in brackets.

Study/plating system tested	Material	Screw diameter (mm)	Insertion torques used (Nm)	Results	
				Pushout testing	Cantilever bending
Kaczmarek *et al.* (21)	Stainless steel	3.5	1.5, 2.5		Not performed
SYN*				2.5 > 1.5 (0.048)	
PLS				1.5 > 2.5 (NS)	
Jung *et al.* (10)					
ARIX	Titanium	2.0	0.2, 0.4, 0.6, 0.8, 1.0	Increased pushout force with increasing torque (*P* = 0.05) for each increase, though increasing failures of screw at highest torques	Not performed
Gallagher *et al.* (19)					
Peri-Loc	Stainless steel	4.5	1.6, 3.2, 4.8, 6.4	Not performed	50% of recommended torque had no significant effect on screw performance when inserted perpendicularly (*P* = 0.892)
Bufkin *et al.* (20)					
PAX	Titanium	3.5	2.5, 3.5	Increase in pushout strength for 3.5 vs 2.5 Nm (*P* < 0.0001)	Not performed
Boudreau *et al.* (7)		3.5	1.5, 2.5, 3.5	Not performed	
NGD	Stainless steel				Increased failure force for 3.5 Nm compared to 1.5 Nm and 2.5 Nm (*P* < 0.05), but no difference between 1.5 and 2.5 Nm
PAX	Titanium				Increased failure force with increased torque (*P* < 0.05)
SYN*	Stainless steel				Increased failure force for 2.5 Nm compared to 1.5 Nm (*P* = 0.008), NS increase comparing 1.5 to 3.5 Nm and NS decrease comparing 2.5 to 3.5 Nm
VOI	Stainless steel				No change with torque
Baroncelli *et al.* (8)		3.5	0.8, 1.5, 2.5, 3.5		No significant difference in bending with different torques except PAX (*P* = 0.03). No *post hoc* analysis, but for PAX: 2.5 > 3.5 > 1.5 > 0.8
Orthomed	Stainless steel			3.5 > 2.5 > 0.8 > 1.5 (NS)	
PAX	Titanium			3.5 > 2.5 > 1.5 > 0.5 (*P* = 0.03)	
SYN*	Stainless steel			3.5 > 2.5 > 1.5 > 0.5 (*P* = 0.03)	
Traumavet	Titanium			3.5 > 2.5 > 1.5 > 0.5 (*P* = 0.04)	
Vet instr	Stainless steel			0.8 > 2.5 > 3.5 > 1.5 (*P* = 0.04)	

ARIX, Atraumatic Rigid Fixation (Jeil Medical Co., South Korea); PAX, Poly Axial (Securos, Germany); Peri-Loc (Smith & Nephew, USA); NGD, New Generation Devices (USA); SYN, Synthes Locking Compression Plate (Synthes, Switzerland); VOI, Veterinary Orthopedic Implants (USA); Orthomed (Orthomed Ltd, UK); Traumavet (Traumavet, Italy); and PLS, Polyaxial Locking Plate System (Aesculap B. Braun Vet Care, Germany).

* All combi-holes.

For pushout testing in full holes (i.e. not combination holes) in stainless steel plates, there was no consistency in results, and no clear impact on performance with changes in torque (8, 21). The three different plate designs being tested produced two different outcomes: no difference in pushout between manufacture-recommended torque and torques larger or smaller than this (19); lower torques were better than higher torques, which in turn still performed better than middle torques (8). For cantilever testing, with the three designs tested, again, no consistency was seen with no impact seen for two experiments (7, 8), and a third showing improved performance with the highest torque, but no difference between lower torques (7).

For stainless steel, combi-hole pushout testing, two studies reported finding significant difference with changes in torque (8). For cantilever testing in combi-holes, one study showed no difference (8) and another showed 2.5 Nm performing significantly better than 1.5 Nm, yet 2.5 Nm was non-significantly better than 3.5 Nm (7). In a second design of a dual hole, 3.5 Nm performed significantly better than both 1.5 and 2.5 Nm, and there was no difference between 1.5 and 2.5 Nm (7).

With titanium plates, there was more consistency with the results, for pushout with more torque improving performance; this was the case for all pushout testing (8, 10, 20). However, with more torque, more construct failures were seen in one study (10). For cantilever testing, a range of outcomes was seen again, with increased torque generating improved performance (7) or no change in performance (8), and using medium torques can perform better than higher torques (8).

## Discussion

Changes in insertion torque do not consistently change the performance of locking screw constructs in different testing modalities reviewed, although for titanium plates in pushout testing, more torque was seen to improve performance. A key variable impacting performance is the engagement of the screw head threads into the locking plate hole (7, 8). This can be influenced by the insertion torque, although it appears that once full engagement is achieved, further torque does not enhance engagement nor significantly increase construct strength. The exception here is titanium plates in pushout testing. With these, and some stainless steel locking plate designs with an interrupted thread, there are increases in pushout strength with more torque after full engagement, achieved through further plastic deformation of the screw threads. This can lead to greater risks of screw head seizing and being difficult to be removed (10, 21). However, against this explanation is the correlation across different systems between a greater number of locking screw head threads and increased pushout strength, independent of the applied torque (8).

### Screw and plate materials

Titanium plates, compared to stainless steel, seem more affected by variations in insertion torque. Of the ten different plating systems tested (19 different instances of plate testing), three were titanium plates and all showed improvements in their failure load in their four pushout tests and one cantilever test with increased torque. However, failure load did not change in two other cantilever tests with variations in torque. With the six stainless steel plates in their 12 tests, in general, neither pushout nor cantilever failure loads changed with more torque, even with the insertion torque increased to 4.4 times the lowest torque (7, 8). Kaczmarek *et al.* did show a difference in pushout load between axially inserted 3.5 mm screws of 2.5 Nm compared to 1.5 Nm (*n* = 4 per torque, *P* = 0.048), inserted into stainless steel 316L plates; however, these were all combi-holes. Any increase in failure load for titanium plates could be due to the ability to plastically deform the titanium screw threads at lower torques compared to the same torque applied to a stainless steel 316L construct and thus increase the thread contact area. The follow-up work by Kaczmarek *et al.* (21) further supports the interpretation that a higher insertion torque improves screw–plate connection by increasing the degree of thread engagement. Their quantitative assessment demonstrated a positive relationship between insertion torque and the proportion of engaged threads in a polyaxial locking system, providing a mechanistic explanation for the observed increases in construct strength under higher torque conditions.

### Construct testing

There were consistent methods used in the pushout testing, where all performed with deformation rates ranging from 1 to 6 mm/min with similar screw constraining techniques. However, only one study inserted the screws into a bone model for pushout testing (8), with the others testing screw and plate constructs in isolation. It is likely that bone failure would occur before locking screw head/screw hole failure (2), thus potentially highlighting most tested failure modes not mimicking clinical failures. All studies used single screw constructs for their tests, and regarding the failure interface being tested, once there is a construct of more than one screw, the plate itself may become the point of loading failure (22, 23). All studies were performed *in vitro*, with all showing awareness of the limitation of isolated *in vitro* screw testing and how it may not be representative of clinical failure, although this consistency in the testing does reduce confounders and allows easier comparisons between results. No studies implemented cyclical loading to mimic *in vivo* plate experiences or considered multiple screws inserted to the same torque being tested at the same time as part of the same construct. Cyclic loading would be especially interesting to investigate given the perspective that screw head locking changes over time with loading and can influence the risk of screw head seizing (9), adding a further reason to avoid overtightening.

The methods of three studies (7, 8, 19) involved cantilever bending, where the force applied to the screw was perpendicular to the shaft – in the direction of the long axis of the plate. All cantilever testing (eight plate designs in ten testing instances) showed no consistent significant change in failure force with increasing torque, except for one plate design. Potentially, cantilever bending is a more realistic failure mode, given that most plating systems are applied on the long axis of bones, with their screws perpendicular to the predominant loading forces during locomotion. These findings further question whether more torque is beneficial to locking plate performance. Two studies tested 3.5 mm diameter screws with the same torque and same plate design; however, they used different testing modes (with different moment arms), making comparisons difficult (7, 8). The loading points on the screws were different, with Baroncelli *et al.* loading at 2 mm from the underside of the plate, recording a value that generated 1.5 mm of deformation (a bend of 37-degrees), whilst Boudreau *et al.* loading at 2.5 mm from the underside, using 0.44 mm of deformation as the end point (corresponding to a 10-degree deflection). Generally, larger values were found with the former testing as a shorter moment arm was tested, and in addition, it was measured to a bigger displacement.

Whilst consistent testing methods, such as pushout and cantilever loading, enable controlled comparisons and minimise experimental variability, they do not fully replicate *in vivo* conditions. Real-world failures are typically multifactorial and often involve cyclic loading, multi-screw constructs and bone–plate interactions. The absence of these factors limits direct clinical translation, although the standardised testing does provide an important baseline for understanding the isolated influence of torque. Future research incorporating cyclic loading and multi-screw configurations could better approximate clinical performance whilst retaining methodological consistency.

### Screw diameters

Three screw diameters were tested amongst the studies reviewed – 2.0, 3.5 and 4.5 mm. However, the core diameters of the 3.5 mm screws varied between 2.4 and 2.9 mm. Variations in core diameter will affect the bending stiffness and could add a confounder to the cantilever tests for comparisons between designs. In both studies of 3.5 mm screws, there was a trend toward increased core diameter correlating with increased bending load (7, 8). For some screws, the locking mechanism is weaker than the screw core bending force required (8) and is likely to be more of the area of interest for future ways to optimise screw design. In addition, one of the tested designs did not have screw threads on the head of the screw, relying on compression for fixation (8), and another relied on locking to occur between the proximal shaft threads and ridges on the bottom half of the screw hole (8); this is not representative of modern locking screw designs, and the results may not be relevant.

### Off-axis insertion

Some of the plate designs included allow for polyaxial locking – allowing up to 20 degrees of off-axis insertion – although only perpendicular insertions in their studies were considered in this review with detailed analysis of the impact of off-axis locking screw orientation with the locking plate being beyond the scope of this article. However, several studies looked at the impact of increasing insertion torque when faced with off-axis insertion, postulating more torque as a remedy to address reduction in cantilever or pushout strength insertion angle deviation causes. Nevertheless, increasing the insertion torque does not compensate for this reduction when compared to perpendicular screw insertion in systems designed for polyaxial locking (10, 19, 21) or designed for fixed-angle locking (19).

For each locking plate design, we postulate that there will be three relationships between torque and performance:

- Undertightened – where the screw head is not fully engaged, reducing the clinical efficacy of the screw through reduced failure loads.
- Correct tightness – where the screw head is fully engaged, optimising failure load, but the screw can still be removed if needed without deformation to either the plate hole or the screw head.
- Overtightened – where more torque is applied after screw head seating, with generally non-significant changes in pushout and cantilever bending loads but with the risk that screw head damage can occur, prohibiting safe removal and potentially distorting the screw hole and plate. In addition, with overtightening, there is a risk of damage to the drive within the screw head (24) and of causing seizing of the screw head within the locking plate hole, making screw removal challenging (6).

Defined upper and lower limits for the ‘correct tightness’ would ensure that the safest and most effective constructs are made. Where manufacturers do make recommendations, undertightening does not consistently reduce the locking performance. Undertightening by approximately 50% of the manufacturer-recommended torque decreased the median bending load in the 3.5 mm locking compression plate (Synthes, Switzerland), but undertightening did not have an effect on 4.5 mm locking compression plates (Peri-Loc; Smith & Nephew, USA) (8, 19). Some implant manufacturers recommend insertion torques for their locking plates, although most did not at the time these studies were performed. Furthermore, the discrete torques selected for comparison in the studies were chosen by their authors for a variety of reasons. Some values were based on the manufacturer’s recommendation, with Gallagher *et al.* doing so and then logically using the recommended value of 3.2 Nm for their 4.5 mm locking screw and plate to define 100%, and testing 50, 150 and 200% of this value to generate a discrete range (19). Bufkin *et al.* also chose their baseline on the manufacturer’s recommendation, but the other torque tested, 3.5 Nm, was chosen ‘as a value that could be achieved in a clinical setting’ (20). Other variables were guided by manufacturers, although some of this advice was qualitative, such as to ensure that screw heads were flush with the bone, estimated to be 2 Nm by the implant company. Other decisions for the chosen torque values were not stated. Some studies acknowledged the lack of direction from some of the manufacturers’ guidance, highlighting that relying on surgeons’ assessments is likely to lead to inconsistent torques (7). Indeed, in controlled non-locking screw testing, the torques applied by surgeons under the same conditions can be extremely varied (25, 26, 27). Thus, for a specific screw design, an acceptable range should be known and targeted with an appropriate torque indicator. This could be indicated with a torque-limited screwdriver that stops driving at a specific value. However, the use of torque limiters needs to be cautious given the variation in the achieved and desired torque with their use, especially when under powered insertion (16, 17). Alternatively, torque indicating screwdrivers could be used to allow for manual control and determination of when the desired quantitative torque was reached.

The authors of all the studies commented on the impact on pull-out force of not engaging all screw threads into the screw hole (7, 8, 10, 19, 20, 21). This effect is magnified with some of the tested screw designs (Traumavet (Traumavet, Italy); New Generation Devices (NGD) (USA) (8); and Synthes Locking Compression Plate (SYN) (Synthes, Switzerland) (21)), where the area of thread engagement increased with more torque. Some locking holes had a portion of the locking screw threads missing to allow the surgeon to alternatively insert a non-locking screw into that area of the plate – ‘combi (combination)-holes’; the missing area allowed flush insertion of a non-locking screw. The absent threaded area in these holes reduces the total thread surface area available for purchase compared to a standard locking hole and is likely to impact on screw failure loads. It would also appear that with combi-holes, more insertion torque enables more thread engagement, given the increased linear advancement of the screw head that more torque achieves, which could explain the increase in performance seen with more insertion torque. However, no direct comparisons were performed between plates that were otherwise the same except for comparing combi- and non-combi-holes within these studies.

### Limitations

The main limitation within the studies is the small number of tests performed for each variable. In total, the results of 247–262 screw insertions were reported (Gallagher *et al.* described between 6 and 11 screw insertions to show an increase in pushout force from 2.5 to 3.5 Nm, although the actual number of tests was not quantitatively reported) in 19 different testing arrangements with 65 torques tested for three screw diameters: 2.0, 3.5 and 4.5 mm. This averages 3.8–4.0 screw tests per condition, highlighting the potential for all studies to be underpowered. Further studies into locking screw performance will need greater sample sizes to produce more powerful results. A further example of the impact from small sample sizes is how the same implant was tested under comparable conditions and produced conflicting results. For pushout tests, the PAX (polyaxial angular stable locking system, Securos, Fiskdale MA) plate had screws inserted to the manufacturer’s recommended torque of 2.5 Nm by Baroncelli *et al.* and by Bufkin *et al.*, respectively, reporting 1,428 N (*n* = 3) and 1,350 N (*n* = 6). When insertion torque was increased to 3.5 Nm, both values increased but remained considerably different: 2,031 N (*n* = 3) and 1,710 N (*n* = 6). For cantilever tests on stainless steel plates, similar methods and the same torques were used on the same screws in two studies for two plate designs. Findings were similar for one of the designs in the two different experiments, although the raw values were considerably different due to differences in the moment arms and the deflection needed for failure load to be determined. However, for the other plate design, more torque (2.5 to 3.5 Nm) significantly increased failure force, and in its other test, this increase in torque non-significantly reduced failure force. The variability within the included studies, with regard to screw diameters, torque ranges, plate designs (including combi- versus standard holes) and testing modalities (especially with regard to moment arms and measured deflection), greatly limits the comparisons possible, and thus the robustness and scope of the conclusions. It highlights the need for consistency with biomechanical testing and the need for standardised protocols when testing insertion torque.

Given the limitations of *in vitro* testing, and the simplification of biomechanical studies in general compared to failure modes *in vivo*, more failure modes are needed to be explored to increase the knowledge transferability of such studies. Finally, there was also a lack of clarity with some of the reporting of results; for example, Gallagher at al. reported that there was no reduction in performance for 50% torque compared to 100% (*P* = 0.892), but without providing the raw values.

## Conclusion

Increased insertion torque for screws inserted perpendicularly into stainless steel plates did not consistently show performance benefits, whilst sometimes worsening failed loads and always increasing the risk of screw seizure. For titanium plates, increased insertion torque generally improved pushout performance, but not consistently cantilever failure force. Biomechanical tests are required to explore the optimum range of torque for locking screw fixation for different materials and to delineate ranges of acceptable torque.

## ICMJE Statement of Interest

JWAF received funding from Invibio (Invibio, Thornton Cleveleys, Lancashire, FY5 4QD, United Kingdom) for performing this review. Invibio had no influence over the methods, data collection, analysis, interpretation or presentation of the results.

## Funding Statement

This work was supported by Invibio (Invibio, Thornton Cleveleys, Lancashire, FY5 4QD, United Kingdom).
